# Medical Practitioners’ Acceptance and Use of AI-Based Clinical Decision Support Systems in Western China: A Mixed-Methods Study

**DOI:** 10.3390/healthcare14081096

**Published:** 2026-04-20

**Authors:** Runping Zhu, Zunbin Huo, Yue Li, Banlinxin Gao, Richard Krever

**Affiliations:** 1 Department of Communication, Beijing Normal—Hong Kong Baptist University, Zhuhai 519087, China; 2 Independent Researcher, Lanzhou 730030, China; henrymengmeng@163.com; 3 School of Journalism and Communication, Lanzhou University, Lanzhou 730000, China; yue.li-45@student.manchester.ac.uk (Y.L.); gaoblx21@lzu.edu.cn (B.G.); 4 Law School, The University of Western Australia, Perth, WA 6009, Australia; rick.krever@uwa.edu.au; 5 African Tax Institute, University of Pretoria, Pretoria 0002, South Africa

**Keywords:** artificial intelligence, clinical decision support systems (CDSS), mixed-methods research, technology acceptance (UTAUT), regional healthcare (western China), social influence, healthcare diagnosis

## Abstract

**Background**: Doctors have made increasing use of artificial intelligence-based clinical decision support systems in recent years in eastern China, but far less so in poorer western China, where hospitals with less access to specialized expert services might be expected to make greater use of such aids. **Methods**: This study of the reasons for lower uptake in the western hospitals focused on a tertiary referral hospital in the capital city of the poorest province in China. Drawing on UTAUT (unified theory of acceptance and use of technology) theoretical literature and previous studies, seven variables most likely to explain the limited adoption of the technology were identified and tested by means of an explanatory sequential mixed-methods study. **Results**: Initial bivariate tests revealed no significant differences across variables; however, multivariate logistic regression identified social influence as the sole statistically significant predictor of adoption willingness. Follow-up structured interviews revealed a surprisingly low awareness of the technology by medical personnel, with very limited deployment. **Conclusions**: The failure to adopt AI diagnosis technology is attributable not to the variables usually cited as factors inhibiting technology adoption but rather the failure of hospital and medical faculty administrators to acquire the technology and train doctors and medical students.

## 1. Introduction

The development of technological innovations in the healthcare industry continues to progress at an accelerating rate. Procedures once limited to major and potentially life-threatening procedures are now completed with straightforward keyhole surgeries, while miniature cameras on journeys through the digestive system can yield far more substantive information than former obtrusive procedures. Progress with tangible technology has been complemented by promising developments on the intangible technology front, particularly in respect of artificial intelligence (AI), for which experts foresee revolutionary potential [1]. Apart from straightforward tasks such as auxiliary medical record recording, clinical nursing, rehabilitation guidance, and automatic guidance, AI is proving to be a game changer in many areas of medical practice [2,3,4] and particularly in terms of clinical diagnosis such as analysis of medical imaging, being able to draw on a wealth of accumulated experience not immediately available to individual doctors [5]. The evidence to date suggests concerns such as perceived data privacy risks or algorithmic bias can be managed to achieve improved outcomes [6].

The AI technology in question, commonly referred to as a clinical decision support system, applies a sophisticated analysis system enhanced by continuous machine learning processes to evaluate a particular patient’s case data in light of a vast dataset to which it has access based on past medical records and make accurate real-time clinical decisions [7]. It can be applied across all forms of medical data diagnosis, such as imaging and radiology for early tumor detection, in cardiology for predictive arrhythmia monitoring, and in pathology for automated histological analysis. In chronic disease management and pharmacology, AI is increasingly utilized for personalized dosage optimization and adverse drug reaction screening.

Unsurprisingly, in China, the technology has seen both tremendous progress and unprecedented levels of state support and sponsorship [8]. The take-up of AI diagnosis should therefore have been predictable, particularly in medical centers located in more rural or remote parts of the country, located far from the specialists and support facilities in the more densely populated and much wealthier eastern provinces. In the east, artificial intelligence-based medical diagnosis support systems are used for purposes such as screening for early-stage lung and esophageal cancers and identification of skin diseases [9,10]. However, its use in the poorer and disadvantaged central and western regions is low [5,11], a phenomenon that seemed odd given what appeared to be the obvious benefits of the technology in these regions [12,13] and less-resourced locales more generally [14].

The low take-up rate prompted the current study, which sought to explore the reasons medical professionals in more remote and poorer locales have been reluctant to make use of AI technology. While the use of AI technology in diagnostic medical practice has been studied elsewhere, and there has been some investigation of its use in rural areas, there is no literature specifically examining the possible reasons for the lower take-up rates in regional hospitals in poorer areas. This paper sought to contribute to closing this gap in the literature.

The site chosen for the study was Lanzhou, the capital of Gansu, a province in the northwest of the country with the unenviable status as the poorest in the nation. More specifically, the study was conducted using medical staff at Gansu Provincial People’s Hospital and with medical students from Lanzhou University Medical School, the primary intern feeder institution for the hospital.

The broader questions of which factors encourage and which inhibit the adoption of AI resources generally and by medical professionals in particular have been the subject of a number of studies globally [15,16,17,18] and in China [19]. However, it is not clear how well the findings transfer from general to the use specifically in particular jurisdictions, as many of the possible factors that impact the adoption of AI technology may be location-specific. Apart from differences in training and practice, there may be more fundamental differences in legal systems, political structures, hospital ownership and management systems, and other environmental features that color any findings. At the same time, a specific case study examination can provide insights into the interaction of factors at one location in the target environment that can subsequently be tested elsewhere.

## 2. Factor Choice and Hypotheses

The adoption of new technology has been driving humanity’s progress, particularly over the course of the 20th and 21st centuries, as cars replaced the horse and buggy, and the telegraph replaced post messages and was in turn pushed to the sidelines by the telephone, radio, television, and the internet. Particularly since the turn of the current century, the speed at which the general public and particular groups are willing to adopt new technology has been a subject of intense interest by scholars whose work in turn is closely watched by the entrepreneurs who hope their technology will be the next target for widespread adoption. The literature draws on a wide array of adoption theories ranging from broad all-encompassing models that bring together multiple theories [20,21,22] through to models that focus on subsets of the many possible concerns of potential adopters [23] such as perceived value [24], perceived risk [25,26], personal innovation awareness [27], and personal choices more general [28]. Other scholars extract individual factors from the multiple-element theories that they believe are most relevant to the technology in question. One factor commonly associated with technology adoption, trust in the efficacy of the technology [29], is seen as particularly relevant to the adoption of medical technology [10,30]. This is an element of the separately identified, broader factor of resistance to adoption of new technology [31], a label that incorporates a range of concerns that include trust in efficacy. While trust is a measurement of positive sentiments, resistance is a measure of negative cognitive opposition.

The current study investigates the factors that might explain the apparent lower take-up of AI-assisted clinical decision support systems in western China, despite the apparent greater need and opportunity for the technology in this region. The two initial steps in the study were the choice of data collection methodology and the identification of factors to be investigated.

To collect a larger dataset for analysis, the study adopted a conventional data collection procedure based on a questionnaire followed by interviews of a small set of respondents. The starting point for the choice of the factors to be included in the questionnaire was a survey of previous studies on the adoption of technology in general and the adoption of medical technology by both medical practitioners and medical students to identify those that proved relevant in similar circumstances. One key assumption, also accepted by other researchers in this area, was the presence of a status quo bias (SQB) theory that posits an expectation that people feel most comfortable staying with known present systems rather than shifting to unknown new systems [32]. While the presumed bias cannot be tested directly, appropriate questions can reveal respondents’ openness to try new systems if made aware of their benefits. A second foundation assumption derives from what is known as the ‘dual-factor theory’, which presumes individuals can be both attracted and repelled by different features at the same time [33,34]. Importantly, factors that might inhibit technology adoption operate independently, and not merely as opposites to enabling factors [35].

The dominant multifactor adoption theory, commonly referred to as the Unified Theory of Technology Adoption and Use (UTAUT) [20] and its expanded successor, UTAUT 2 [22], were used to collect an initial set of potentially relevant factors, which were then compared to the factors used in other medical adoption studies. Some of these, such as a privacy concern that data could be collected and exploited by large corporations providing clinical decision support systems [36], were clearly location-specific factors that find little resonance in public clinics in western China using state-provided software. Others that looked potentially relevant were considered if they had an impact on willingness to use the new technology.

The list of potential factors was whittled down to seven that appeared to have the largest impact on views about clinical decision support systems and were most likely to be transferable to clinicians in western China. Three of these were drawn from the UTAUT theory and four others from other literature on technology adoption, particularly studies on adoption in medical environments. Multiple questions were then devised to test the impact of each factor.

The first factor, drawn from the UTAUT theory, was performance expectations, defined in the UTAUT model as ‘the degree to which an individual believes that the application of the technique will help him or her to achieve job performance’ [20]. It has been seen that physicians will be motivated to use and embrace new technologies if they perceive them to be more effective and beneficial to their clinical practice than existing methods [9,10].

A second factor, also drawn from the UTAUT theory, is expectation of effort, defined in the original articulation of the model as ‘the ease with which the system is used’ [20]. Again, it has been shown that perceptions regarding the ease-of-use correlate directly with intention to use new technology [37,38], and an easy-to-use system is more likely to be used than a complex one [39].

The third factor taken from the UTAUT theory that appears to have an impact on intention to adopt new technology by medical personnel has been labeled social influence, meaning a user’s perception of the views and actions of a circle of potential influences comprising peers, seniors, mentors, and others relevant to the situation in which the technology would be used [20]. In different studies, social influence has been shown to be both important in promoting physician adoption of healthcare information technology HIT [39] and unimportant in this respect [40]. A number of questions were devised to determine how the opinions of people in doctors’ social and professional circles might have influenced their intentions to use AI-based clinical decision support systems.

The fourth and fifth factors tested in the study together comprise potential users’ trust in AI-based support technology. The factor is bifurcated into two elements, ‘initial trust’ [41,42], that is, trust when confronted with new technology, and elemental trust, which is confidence that the technology will provide reliable, accurate, and safe results based on general knowledge, experience, personality, and broader worldview of technology. Initial trust plays a crucial role when it comes to new technologies, as perceptions of risk and uncertainty need to be overcome in order to develop a willingness to use these technologies [43]. Initial trust has been found to play a mediating role in the willingness to adopt technologies in a variety of environments [9,43,44]. The companion element of trust in the elements of the system may be particularly important for AI medical technology [45], given the ‘black-box’ nature of the algorithms and processes used in clinical decision support systems that are inherently invisible to the users.

The sixth factor examined as a mediator for clinical decision support system take-up, consideration of risk, has dual medico-legal limbs [10]. For example, the medical risks considered by doctors relate to the risk to the patient from a clinical decision support system that generated a false-negative conclusion. The legal risk is the risk faced personally by the doctor who relies on the clinical decision support system when providing advice to the patient. While the legal risk for a practitioner who relies on a clinical decision support system made available by his or her institution is uncertain under current Chinese law [44], the unknown risk may have a negative influence on physicians’ willingness to use the new technology.

The seventh and final factor tested in the study is the perceived threat to occupational autonomy or even replacement, an identified determinant of willingness to use an AI-based support system [46,47]. Practitioners may be concerned that AI will infringe upon their professional discretion, erode their decision-making authority, or ultimately replace human expertise. The threat transcends simple job security; it concerns the fundamental role of a doctor, where an algorithmic ‘black box’ might dictate clinical responses, reducing the physician to a mere executor of software-generated protocols.

Our study of technology adoption led us to five hypotheses:

**H1.**  *Expectations of work performance improvement, such as increased speed, accuracy, and productivity, and reduced workload, will be more likely to generate an intent to use AI-based support.*

**H2.**  *If physicians perceive AI assistance to be simple and easy to operate, they would be more inclined to use it in their medical practice.*

**H3.**  *There would be a positive correlation between social influence, particularly adoption by colleagues, senior staff, or others in doctors’ social groups, and the doctors’ behavior and greater use of AI.*

**H4.**  *Four factors would have a negative impact on physicians’ willingness to use the new technology: lack of initial trust in new technology; lack of elemental trust in an AI-based system due to inability to follow its reasoning processes; concern over possible risks from use of the software; and concern over the threats it poses to professional autonomy and tenure security [48].*

Aside from the external factors shown in some studies to have an impact on technology take-up, the study collected demographic details to investigate whether two demographic features, age and gender, might have an impact on physicians’ willingness to use an AI-based support system. Rather than select arbitrary age points to distinguish doctors by generation, the study consolidated age with professional status and compared different age groups of practicing doctors with medical students and those already in practice. This approach extended the age factor beyond that adopted in studies of healthcare take-up of artificial intelligence in rural settings [49]. No assumptions were made with respect to the influence of gender on technology acceptance.

**H5.**  *Consistent with findings in other locales [50], the younger generation would be more open to incorporating AI-based clinical support into their medical procedures.*

## 3. Survey Process

The study employed an explanatory sequential mixed-methods design, conducted in two distinct, non-overlapping phases. In the first phase, a quantitative study was implemented to identify statistical correlations between the seven theoretical variables and adoption willingness. Following the emergence of unexpected non-significant results in the quantitative analysis, a second qualitative phase was executed. This sequential approach allowed for the qualitative interviews to act as a diagnostic tool, providing a deeper explanation for the quantitative null results.

Data for the study were collected in January 2024 using a convenience sampling strategy, targeting healthcare professionals at four hospitals as well as medical students in Lanzhou, Gansu Province. The four hospitals were the Yuzhong County People’s Hospital, the First and Second Hospitals of Lanzhou University, and Gansu Provincial Hospital. These last three of these dominate the local healthcare landscape, attracting 75 per cent of the total patient visits due to their advanced clinical expertise.

The researchers realized the targeted population would be difficult to reach due to demanding clinical schedules and restricted institutional access. To mitigate these barriers, the survey was distributed via an online platform to accessible medical staff across various departments. Access was facilitated through a top-down institutional approach, leveraging the professional networks of senior clinicians at leading tertiary hospitals and medical universities. By targeting Gansu Provincial People’s Hospital—a leading tertiary institution—and Lanzhou University Medical School—the province’s primary feeder for clinical talent—the sample captures the critical stakeholders within the regional medical hierarchy. The demographic distribution (detailed in Table 1) reflects a comprehensive cross-section of clinical experience, ranging from medical interns to senior practitioners with over 25 years of employment. This diversity across professional lifecycle stages, combined with broad coverage of clinical departments, ensures that the sample is representative of the frontline medical personnel who are potential users of AI-based clinical decision support systems.

The questionnaire posed 34 questions concerning the seven factors investigated in this study and demographic variables. Questionnaire access was provided to a total of 200 respondents, of whom 189 submitted completed questionnaires. Twenty-three of the responses were excluded (for example, invariant response patterns with the same scale point for all items or missing data), leaving 166 fully valid responses, a response rate of 83%. The survey group provided a broad coverage of the desired demographic targets, albeit with a strong female weighting across all age groups.

The questionnaire contained four demographic questions (gender, age, years of work, and hospital role) and 30 questions measuring seven independent technology adoption factors, alongside queries measuring willingness to use the technology, adopted with appropriate modifications from previous studies that focused on individual factors [29,46,51,52,53]. Five questions were used for performance expectations, three for the ease of use factor, four for the social influence factor, four for the initial trust factor, three for ongoing trust, four for perceived risk, and four for perceived threat. All 30 questions were tied to a five-point Likert scale suite of responses (1 = strongly disagree, 5 = strongly agree).

As this study focuses on the utilization patterns among medical students and practitioners, the minimum age was set at 18, with the 18–25 age cohort capturing the undergraduate medical education phase. The 26–30 bracket accounts for the extended professional training cycles characteristic of medical education, such as clinical internships and postgraduate residency, serving as a transition from student to physician. Participants aged 31 and above are primarily practicing doctors, making data from this group highly significant for the assessment of willingness to adopt the new technology. The 31–40 and 41–50 groups are categorized in 10-year intervals to examine intergenerational cognitive disparities regarding new technologies. Finally, individuals over 50 are aggregated into a single category without further subdivision, as their exposure in China to nascent technologies has been historically limited.

## 4. Empirical Findings

The questionnaire responses were subject to a binary logistic regression model with the use of SPSS 27, the methodology widely used in public health correlation and pathology studies, and which is mostly used to deal with variables of dichotomous or multi-categorical data samples [54,55], as well as a conventional *t*-test methodology to determine which of the possible correlations identified in the study were statistically significant. The analysis was based on seven independent variables (factors that might affect willingness or intent to adopt AI technology) and a single dependent variable (the willingness or intent to adopt this technology). For the empirical analysis, the 30 Likert-scale items were treated as interval data by calculating composite mean scores for each of the seven theoretical constructs, Table 2.

The reliability of the questionnaire was measured against Cronbach’s alpha measurement and its validity using the KMO test and Bartlett spherical test, Table 3. The results indicated the questionnaire had strong reliability and validity, outcomes confirmed using two further checks, the Hosmer and Lemeshow tests.

With the combination of a high rate of valid responses and empirical confirmation that the questionnaire was robust, we anticipated a wealth of data to confirm or refute our seven operative hypotheses. Quite surprisingly, the *t*-test findings, testing the statistical significance of the apparent correlations or lack thereof between views on the seven factors and willingness to use an artificial intelligence support system, showed that not one of the seven factors generated a statistically significant outcome. The outcome was similar when responses were broken down by different demographic features—that is, there were no statistically significant correlations when each variable was examined by male and female respondents separately, different age groups, and so on.

The data were also subject to binary logistic regression, which measures the relative strengths of possible connections between each variable and practitioners’ willingness to use an AI clinical decision support system. For the most part, the results echoed the *t*-test findings, with six of the seven variables having no significant effect on the willingness to use the AI clinical decision support system. The one exception to the general findings was in respect of social influence, which did appear to have a significant impact on willingness to use the artificial intelligence clinical decision support system. The different outcomes between the *t*-test and binary logistic regression analysis reflect the fact that in a *t*-test, each factor is examined separately, while the regression analysis can find correlations between factors and dependent variables relative to all other factors.

The regression analysis results are presented in Table 4. The dependent variable is denoted as ‘M act intent,’ representing the medical practitioners’ actual behavioral intention to adopt AI-based clinical decision support systems. This was determined using a 5-point Likert scale ranging from no intention to full intention. While the preliminary *t*-tests suggested a lack of individual significance across all seven constructs, the inclusion of all variables in a logistic regression model provided a more nuanced view. Once other factors were controlled for, social influence (*p* = 0.027) emerged as a significant determinant. This suggests that in the specific context of regional China, the perceived expectations of peers and authorities outweigh individual perceptions of effort or performance.

## 5. Qualitative Findings

The starting point for the research project was an acceptance of findings that doctors in the poorer and more remote west of China make less use of modern AI diagnostic tools than their counterparts in the wealthier and better-resourced east of the country, notwithstanding the apparent greater need for, or potential benefit from, tools that can augment resources in the west. It was, thus, surprising that the empirical study could find no links between variables and behavior for six factors that have been found to be connected to the adoption of new technology generally and new medical technology in particular in other environments.

In parallel with the analysis of survey data, qualitative follow-up interviews were carried out with a smaller number of participants following the conclusion of the initial survey. Interviews took place at a teaching tertiary hospital in the capital city of the province, home to the province’s university medical faculties and the province’s better-equipped medical facilities. The study thus portrays a ‘best-case’ scenario for the adoption of new technology. Findings on factors behind limited adoption would automatically apply to the rest of the province, and the findings might be unrepresentative only to the extent that the study showed a positive uptake of new technology in Lanzhou institutions. However, as a goal of the study was to provide contextual insight rather than broad statistical extrapolation, the sample provides a representative snapshot of the regional medical landscape.

A total of 16 interviews were conducted, with participants selected wholly at random from across the department. Questions were posed from a pre-determined interview outline, and responses were tabulated and set out in a tabular form for analysis.

Importantly, the interviews were conducted before the regression analysis of survey data had been completed. As a consequence, the interviewers did not know the survey would disprove six of the seven operative hypotheses assumed by the researchers. Therefore, this interview did not focus on links between the factors and use of clinical decision support systems, but more broadly looked at participants’ specific experiences using artificial intelligence aids and their understanding and general feelings about the use of artificial intelligence, in addition to information about the possible nexus between the seven variables investigated and the adoption of AI-based decision assistance. Since the quantitative outcome of the initial survey yielded no convincing explanation for the low take-up rate of AI clinical decision support systems, the results of subsequent interviews proved crucial to the conclusions reached in the study.

The interview structure and content followed the precedent adopted in an earlier study of AI adoption by medical practitioners [36] and the Castillo-Montoya Interview Protocol Refinement framework commonly used for qualitative interview studies [56]. Question themes parallelled those in the questionnaire, so interviewees could have an opportunity to expand on their views beyond the Likert scale responses. Prior to the interviews, the researchers sent the interview outline to the interviewees. Interviews were recorded, and the recording began after an explanation and consent for the recording.

Two independent coders performed the initial categorization of interview transcripts. Inter-coder reliability was assessed using Scott’s Pi, which yielded a coefficient of 0.86, indicating a high level of inter-coder consistency. Any minor discrepancies were resolved through collective deliberation to reach a final consensus. Analysis of qualitative data was based on a directed content analysis approach that followed a structured deductive process in which interview transcripts were systematically mapped onto the pre-defined theoretical constructs identified in the quantitative phase.

Although the links between all factors bar one and an inclination to use or shun clinical decision support systems were neither statistically significant nor identifiable using regression analysis, personal interviews suggested two possible explanations. The first is that all factors were relevant, but in a cumulative fashion, so that six of the seven would not record a persuasive link on their own, but when taken together, would lead to reluctance to adopt the new technology. The second is that hospital administrators and university medical teachers have made relatively little effort to introduce modern AI-based clinical decision support systems into use in the hospital or training for doctors, so respondents’ conclusions and sentiments are largely rooted in limited awareness about the actual operation of AI systems. Evidence from the follow-up interviews supports both of these hypotheses.

The limited awareness explanation was supported by the observation by most of the practitioner respondents that their knowledge of AI support systems came not from experience or information provided by their hospitals but rather through outside sources, particularly internet sites. The importance of healthcare managers and their institutions in the adoption of AI has been emphasized elsewhere [57,58], and these findings reinforce conclusions on the limited adoption in the case study featured in this article. The conclusions concerning the importance of management support are also consistent with findings outside the healthcare sector [59,60]. The use of AI support systems was also not incorporated into the curriculum of medical students whose classes touched upon the subject but included no instruction or actual illustration, leaving a group that might have been expected to be more enthusiastic about computer-based assistance largely in the dark about the potential of AI support for clinical decisions.

While never a primary issue for interviewees in the sense of being a deal-breaker, respondents all noted some concern about the technology. A fundamental concern was whether AI-based systems worked—the view was expressed that AI could not be as accurate as a doctor’s assessment because the computer could not consider aspects such as the patient’s psychology. Other concerns included the security of the system in terms of protecting patient information from leakage to outsiders and operational reliability—if diagnosis was delegated to the software, no decisions could be made when the power went out, or the network crashed. The latter point was mentioned several times, likely a consequence of the previous year’s incident when the optical fiber of the hospital was cut by the construction crew, paralyzing the hospital’s work as it became impossible to retrieve patients’ registration or prescribe medicine. The perceived impact of the incident was profound, and it contributed to broader trust concerns with AI-based technology.

The explanation for the almost complete absence of concern about liability or risk from the adoption of AI systems offered by one doctor is the lack of knowledge about a system not in general use or even in the process of adoption in most instances; it was impossible to develop concerns about an almost wholly unknown system. The limited concern contrasts with the attitudes of physicians outside China [61].

An issue that was consistently dismissed by respondents as a concern was that of occupational threat. The issue was expressed not in negative terms, as a shortcoming of technology, but rather as an affirmation of the importance of ongoing clinical evaluation by doctors as a necessary complement to any AI-based diagnosis. Technology, doctors explained, could look at evidence provided to the system, but human evaluation was needed to evaluate other clues, such as patients’ expressions, movements, eyes, and other evidence of symptoms. While variations in this point recurred, all were based on speculation as to how AI-based diagnosis worked, with no evidence of understanding of its operation in practice.

A separate but related subject of study is patient skepticism or resistance to AI and preference for human advice [19,34,62]. Medical professionals aware of this skepticism would no doubt take it into account when deciding if and when they turn to AI for assistance [63]. However, it is not clear to what extent patients would be aware of the details of the medical diagnosis process in their case and pass on their views of the process to the professionals treating them.

One issue that was not a concern to any interviewees was the ease with which use of AI clinical decision support systems could be mastered, or the challenge of learning a new system would entail. Doctors pointed out that they use complex instruments daily in medical practice, and as new techniques and equipment become available, they master them as one of the aspects of their chosen careers. None expressed concern over learning to operate the new system, which could dissuade them from incorporating AI-based support in their medical practice.

Finally, interviews confirmed and reinforced the regression analysis finding of a positive link between social influence and support for the adoption of AI clinical diagnosis tools. Doctors noted that most patients trust modern medical technology generally and would likely welcome it and be reassured if they knew the latest technology was being used to diagnose their illness. If the technology were made available to them and their colleagues adopted it, they would quickly follow suit to ensure colleagues and patients saw them as part of the modern care-giving regime.

The most important link between the qualitative and quantitative findings is the possible explanation for the lack of statistical correlation for independent factors and the dependent variable of intent to adopt AI technology. The qualitative data suggest the quantitative findings reflect a pre-adoption state of technological invisibility. As university instructors and hospital administrators have not yet formally introduced AI-based clinical decision support systems, participants were evaluating these constructs in a vacuum of practical experience. Consequently, the quantitative ‘null results’ are qualitatively explained as a consequence of institutional inertia rather than user indifference or resistance.

## 6. Limitations

Interviews are by their nature not anonymous, at least at the time the information is provided, a format that introduces a risk of social desirability bias as respondents provide answers they believe will conform to social norms or expectations.

A possible limitation of the study was the limited pool of respondents (chosen from one hospital and one medical school). While this was likely to be sufficient for an understanding of medical technology adoption attitudes in the capital city of a poor province, the results may not be reflective of the remainder of the poorer parts of the country.

A more significant limitation was the fact that the study focused on the traditional subject of technology user adoption studies, the users themselves, while in this case, the preliminary crux of resistance to adoption lies with the administrators who could make the technology available. The study reveals an opportunity for intervention by higher authorities to achieve modernization and possible clinical and efficiency benefits. The focus on practitioner views as the primary factor for the adoption of technology had arguably initially obscured the need to consider the role of institutional factors outside the scope of individual practitioners.

Finally, the study is a cross-sectional investigation, meaning all data relate to a limited time period. Had the study been carried out over different time periods and the availability of, or adoption of an AI-based clinical diagnosis support system changed over the periods, the study may have yielded different findings and conclusions.

## 7. Conclusions

The study sought to investigate the reasons for the lack of adoption and use of AI-based clinical diagnosis tools in the poorest province of China, a region in which the assistance of technology could be expected to be particularly useful. The absence of this medical assistance clearly exacerbates the rural–urban health equity gap.

The study tested current and soon-to-be doctors’ views about the adoption of AI clinical decision support systems using factors shown in both the theoretical literature and previous studies to be relevant to technology adoption. The study could not confirm or refute the five hypotheses investigated and revealed little about the impact of users’ views on the adoption of new technology. Rather, it showed that conventional technology adoption factors may be irrelevant when potential users lack basic awareness that technology even exists, and adoption was not an option for them.

The initial acquisition and deployment of new technology in this case rests not with the potential users but rather with a much smaller group of hospital and, in the case of medical students, university administrators who, thus far, have not moved in line with their counterparts in the east of the country. In contrast to studies that find a limited adoption of AI may be traced to technical deployment bottlenecks [64], our study in regional Gansu suggests an even more foundational hurdle is the complete absence of institutional introduction and formal training.

The findings support studies that suggest education is crucial to the take-up of AI clinical decision support systems [64], as is an optimal implementation program [65,66]. Specifically, hospital administrators should prioritize the acquisition of appropriate AI software and corresponding hardware for the operation of the AI, and hospitals and universities should work together to ensure new doctors and current medical professionals are fully trained in all aspects of AI application in clinical diagnosis.

One factor not investigated in the study is practitioners’ consideration of patients’ concerns over the adoption of new technology, a potential factor [67], including in China, where there is some evidence of patient distrust in AI medical systems [63,68,69]. Future studies of acceptance of AI in clinical diagnosis might also break down data to reveal possible differences between different areas of medical practice, as some areas, such as radiology, are more likely to be affected by the technology than others [70,71].

## Figures and Tables

**Table 1:** Demographic details of respondents (*n* = 166).

Variable	Category	Frequency	Percentage
Gender	Male	44	26.50
	Female	122	73.50
Age group	18–25	38	22.90
	26–30	28	16.90
	31–40	39	23.50
	41–50	52	31.30
	>50	9	5.40
Years of employment as a doctor	Medical students	33	19.88
	<5	30	18.07
	5–15	37	22.29
	15–20	12	7.23
	20–25	22	13.25
	>25	32	19.28
Hospital role	Clinical (directly diagnose and treat patients)	128	77.1
	Non-clinical (provide treatment and administrative support)	38	22.9
Total		166	100

**Table 2:** *t*-test of intenders’ and non-intenders’ means.

Variable	Mean (Intenders, *n* = 133)	Mean (Non-Intenders, *n* = 33)	t-Value	*p*-Value
Performance Expectancy	4.25	4.16	0.62	0.537
Effort Expectancy	3.91	3.75	0.94	0.349
Social Impact	4.07	3.78	1.92	0.056
Initial Trust	4.11	4.06	0.32	0.747
Elemental Trust	4.04	3.87	1.15	0.253
Perceived Risk	1.86	1.9	−0.3	0.762
Perceived Threat	2.17	2.28	−0.66	0.509

**Table 3:** Reliability statistics.

Category	Cronbach’s Alpha	Cronbach’s Alpha Based on Standardized Items	N of Items
Total Sample	0.951	0.957	34
Key Variables (Influencing Factors)	0.966	0.968	30

**Table 4:** Regression analysis findings.

		B	S.E.	Wald	df	Sig.	Exp (B)	95% C.I. of EXP (B)	
								Lower Limit	Upper Limit
Step 1a	Performance expectations	−0.097	0.489	0.039	1	0.843	0.908	0.348	2.365
	Expectation of effort	−0.031	0.327	0.009	1	0.923	0.969	0.510	1.840
	Social influence	1.154	0.522	4.894	1	0.027	3.172	1.141	8.818
	General trust tendencies	0.329	0.460	0.510	1	0.475	1.389	0.564	3.426
	Initial trust perception	−1.265	0.709	3.179	1	0.075	0.282	0.070	1.134
	Perceived risk	0.089	0.397	0.051	1	0.822	1.093	0.503	2.378
	Perceived threats	−0.012	0.277	0.002	1	0.965	0.988	0.574	1.701
	M act intent	0.231	0.375	0.378	1	0.539	1.260	0.603	2.629
	constant	0.182	2.239	0.007	1	0.935	1.200		

Note: B = coefficient/beta. S.E. = standard error. Walds = Wald chi-square statistic. df = degrees of freedom. Sig. = significance/*p*-value. Exp (B) = odds ratio. C.I. = confidence interval. EXP (B) = exponentiated value of B (odds ratio).

## Data Availability

Data and materials are available from the data management author, Dr. Runping Zhu (runping.zhu@outlook.com), upon reasonable request. The data are not publicly available due to privacy and ethical restrictions.
